# Double Negative T Regulatory Cells: An Emerging Paradigm Shift in Reproductive Immune Tolerance?

**DOI:** 10.3389/fimmu.2022.886645

**Published:** 2022-07-01

**Authors:** Enitome E. Bafor, Julio C. Valencia, Howard A. Young

**Affiliations:** Cancer Innovation Laboratory, Center for Cancer Research, National Cancer Institute, Frederick, MD, United States

**Keywords:** double negative T cells, double negative T regulatory cells, ovulation, endometrium, reproduction, immune tolerance, implantation, reproductive immunology

## Abstract

Immune regulation of female reproductive function plays a crucial role in fertility, as alterations in the relationship between immune and reproductive processes result in autoimmune subfertility or infertility. The breakdown of immune tolerance leads to ovulation dysfunction, implantation failure, and pregnancy loss. In this regard, immune cells with regulatory activities are essential to restore self-tolerance. Apart from regulatory T cells, double negative T regulatory cells (DNTregs) characterized by TCRαβ^+^/γδ^+^CD3^+^CD4^–^CD8^–^ (and negative for natural killer cell markers) are emerging as effector cells capable of mediating immune tolerance in the female reproductive system. DNTregs are present in the female reproductive tract of humans and murine models. However, their full potential as immune regulators is evolving, and studies so far indicate that DNTregs exhibit features that can also maintain tolerance in the female reproductive microenvironment. This review describes recent progress on the presence, role and mechanisms of DNTregs in the female reproductive system immune regulation and tolerance. In addition, we address how DNTregs can potentially provide a paradigm shift from the known roles of conventional regulatory T cells and immune tolerance by maintaining and restoring balance in the reproductive microenvironment of female fertility.

## Introduction

Immune tolerance during the female reproductive cycle and pregnancy is required for fertility. Successful ovulation, fertilization, and pregnancy rely on an efficient regulatory mechanism that prevents immune responses to sperm cells, oocytes, and placental cells, which express various antigens (1–6). Several female pathologies are linked to immune tolerance dysfunctions, including preeclampsia, recurrent miscarriages, and autoimmune ovarian failure. Pre-eclampsia, which accounts for about 20% of maternal mortality, is associated with a dominant Th1 inflammatory response resulting from impaired function of cells with regulatory activities (7–10). Recurrent miscarriages, which affect about 1-5% of women, are associated with reduced regulatory T cells (11, 12). More recently, autoimmune ovarian dysfunction, a disorder affecting female patients with autoimmune disorders, has been associated with immune tolerance alterations (13–15). These disorders highlight that an imbalance in the immune tolerance equilibrium in the female reproductive system (FRS) can cause severe gynecological and obstetrical complications.

Suppression of immune responses by cells with regulatory activities is one of the central mechanisms for induction and maintenance of self-tolerance (16). CD4^+^CD25^+^ T regulatory cells (CD4^+^Tregs) are the most extensively studied suppressor cells regarding reproductive failure and reproductive immune tolerance (17–19). Furthermore, estrogen and seminal fluid drive the expansion of CD4^+^Tregs, enhance their suppressive function (11, 21, 22), and regulate their activities in the FRS. However, recent data finds that a subset of T cells with unique potent regulatory functions and dominance in the reproductive system (20, 21), known as the double negative T regulatory cells (DNTregs), also play crucial roles in regulating immune responses (21–24). This current review focuses on understanding the characteristics and function of DNTregs as potential regulators of reproductive female immune tolerance and their potential as a novel therapeutic option for reproductive failure, including those associated with autoimmunity. Specific details of DNTregs on non-reproductive autoimmune conditions such as graft versus host disease (GvHD) and cancer are covered elsewhere (25–28). We will provide the context for the interplay of DNTregs in reproductive function. We will also discuss and compare the presence, function, and relevance of CD4^+^Tregs and DNTregs in reproductive immune tolerance. Finally, this review will address how DNTregs represent a potentially viable therapeutic option in female reproductive disorders, including autoimmune conditions.

## Immune Regulation in the Reproductive Cycle – A Tightly Controlled Physiologic “Inflammatory” Process

Immune cells modulate several aspects of the female reproductive function, including folliculogenesis, ovulation, implantation, pregnancy, and labor (29, 30). At distinct points in the reproductive cycle, different immune cells are identified throughout the FRS. Immune cells occur in high frequencies within the upper FRS (the ovaries, oviduct, uterus, and endocervix) and occur less in the lower FRS (ectocervix and vagina) (31). The FRS is an exceptional immunological site, and the distribution of immune cells facilitates tolerance to allogeneic sperm and the semi-allogeneic fetus and placenta (11, 32, 33).

During the reproductive cycle, steroid hormones and immune cells regulate ovulation, fertilization, and implantation in a cyclic fashion and tandem (34–36). Fluctuations of estradiol and progesterone drive relevant processes within the uterus and ovary, including cyclic recruitment of immune cells (37). Within the FRS, immune cells are primarily under ovarian hormone regulation (31, 36, 38) and contribute to the modulation of the reproductive cycle and fertility (39). For instance, T cells (and immune cells in general) express sex-steroid receptors (40–43), and the high infiltration of CD8^+^ T cells into the regressing corpus luteum (CL) coupled with the expression of cytolytic proteins corresponds to decreased progesterone and estradiol concentrations during the period of luteal regression in the ovaries (44). Furthermore, alterations in the immune system affect ovarian function in both animals and humans (45). For instance, blockade of gonadotropin-releasing hormone (GnRH), which centrally regulates the hypothalamic-pituitary-ovarian axis (46), decreases regulatory T cells’ proliferation and thymic mass (47).

Moreover, estrogen deficiency corresponds with increased peripheral cytotoxic T cells and CD8^+^/CD4^+^ T cell ratios. Furthermore, the rise in circulating estrogen levels is associated with increased CD4^+^Treg populations (48). These findings indicate that the reproductive hormones tightly regulate immunological changes in the ovary and endometrium (49).

## Double Negative T Cells: Origin and Function

Most mature αβ T cell receptor (TCR)^+^-T cells in normal mice and humans express either the CD4 or CD8 coreceptor molecules. However, approximately 1–5% of the peripheral T cell population that expresses CD3 but neither the CD4 nor the CD8 coreceptor is termed, CD4 and CD8 double-negative T (DNT) cells. DNT cells were identified in spleen cells from irradiated mice over 40 years ago, but at the time, they were referred to as natural suppressor cells (50, 51) with a null phenotype in neonatal mice (52). In 1984 Oseroff et al. reported that DNT cells do not express the T cell marker CD90/Thy-1, the surface immunoglobulin (Ig), the myeloid marker CD11b/MAC1, the macrophage marker F4/80, or the monocyte specific esterases (52). Their study reported that DNT cells were similar to natural killer (NK) cells based on their combined lack of antigen-specificity and coreceptor CD4 and CD8 molecules (52, 53). However, later reports found that DNT cells inhibited T cell response to alloantigen (54, 55) in an antigen-specific manner (24). Similarly, early reports on this subject suggested that the suppressive function of DNT cells may not depend on proliferation, as suppression was maintained after exposure to high levels of radiation (56). This finding partly explained why DNT cells exert potent suppressive functions despite low numbers. Furthermore, early research also suggested that these unique cells were dependent on T cells in the spleen (50) and that their function becomes dominant when CD4^+^, CD8^+^ T cells, and B lymphocytes are unable to function effectively (56). However, DNT cells with regulatory functions (DNTregs) were not classified as such until 2000 (24).

Naturally occurring T cell maturation and differentiation into CD4^+^, CD8^+^, and CD4^+^Tregs require thymic development (57, 58) though peripheral or induced CD4^+^Tregs can develop from peripheral naïve conventional T cells (59). However, the origin of DNTregs is still a matter of debate. Several reports suggest that DNT cells originate from the thymus either as DNT cells or from single positive (SP) T cells under regulation by sex steroids (21, 60). However, researchers also found that large DNT cell populations can develop, mature, and gain regulatory function in the absence of a thymus or outside the thymic microenvironment (61), just like the peripheral CD4^+^Tregs. In support of the latter, other studies found that DNT cells can develop and mature in the bone marrow (62), liver (63), nasal-associated lymphoid tissues (NALTs) (64), and, as concerns this review, in the FRS (20). Furthermore, studies that suggest CD4^+^ or CD8^+^ precursors for DNT cell maturation indicate a requirement for the thymus at least for their initial development (65–67). The conclusions for DNTs' origin from CD8^+^-derived DNT cells were based on the observation that antigen encounter decreases CD8^+^ populations and increases DNT cell numbers *in vitro* (68, 69) or *in vivo* (70, 71). However, recent *in vivo* data revealed that the development and function of DNT cells could occur in the absence of CD8^+^ cells, indicating that DNT cells can develop from a cell lineage independent of CD8 expression (61), consistent with an earlier report (52). The increase in DNT cells in the study describing decreased CD8^+^ populations upon antigen encounter may have been due to direct activation and expansion of a pre-existing population of DNT cells. Other studies propose that DNT cells in *lpr* mice (which have null allele for *Fas* (CD95) (72)) may occur from CD8^+^T cell precursors in the periphery (73, 74) and that DNTs may also occur from CD4^+^ T cell precursors on stimulation of CD4^+^T with allogeneic DCs in the presence of IL-15 or IL-2 (22). Taken together, DNT cells may develop from a separate lineage. However, there may be some degree of peripheral differentiation from peripheral single positive T cells, and further studies are required to determine their precise origins.

### Phenotypic Characterization of DNTs

Total DNT cells comprise 1-5% of total peripheral T cells in mice and humans, and their functions and phenotype have been characterized (24, 75, 76). Based on the expression of NK cell markers, DNT cells can be divided further into two subpopulations: NK^+^DNT cells, referred to as NKT cells (67, 77), and NK^−^DNT cells, referred to as DNT cells (24, 75, 78). Reports suggest that DNT cells exist as cytolytic or regulatory subsets (DNTregs) and bear either the TCRγδ or TCRαβ repertoire (79–82). Though some reports show that DNT TCRαβ cells exhibit regulatory effects and are characterized as TCRαβ^+^CD3^+^CD4^-^CD8^-^ NK1.1^-^T cells (24, 75), other reports suggest that DNT cells expressing TCRγδ also exhibit regulatory potential (21, 83). Furthermore, mature peripheral DNTregs differ from bone marrow-derived DN natural suppressor T cells that express NK1.1 (84–87).

CD4^+^Tregs consist of naturally occurring Tregs (nTregs or thymic Tregs) and inducible or peripheral Tregs (iTregs or pTregs). By contrast, current evidence indicates that total DNT cells are heterogeneous. By utilizing single-cell RNA sequencing, flow cytometry, and qPCR to analyze total DNT cells from C57BL/6 mouse spleens, a recent study investigated naïve and activated total DNT populations (nDNT and aDNT, respectively) (88). The study found high gene expression of FasL (*Fasl)*, granzyme b *(Gzmb)*, interferon-gamma (*Ifng)*, killer cell lectin-like receptor D1 (*Klrd1)*, killer cell lectin-like receptor C1 (*Klrc1)*, and killer cell lectin-like receptor K1 (*Klrk1)* in nDNT or aDNT subgroups. These findings indicated that these cells exert regulatory functions (88) and may represent the DNTreg phenotype. The study demonstrated that naïve populations significantly expressed the Ikaros family zinc finger 2 (*Ikzf2*) and lymphocyte antigen 6 complex locus C2 (*Ly6c2*) genes (88). The transcription factor IKZF2 is expressed by T cells undergoing central and peripheral tolerance (89). Beyond regulating IL-2 production by Tregs, studies indicate that *Ikzf2* is also required to stabilize the suppressive phenotype in Foxp3^+^Treg populations (90). Though the Ly-6C protein is known to recruit macrophages in murine liver fibrosis (91), it is essential for the development of naïve and activated DNT cells, particularly the DNT subsets with regulatory functions. For clarity, the study associated the ‘cytotoxic’ DNT subsets with regulatory function. It is, however, unclear if this ‘cytotoxic’ phenotype consists of a heterogeneous population with effector ‘cytotoxic’ DNTs that drive disease pathology and a regulatory ‘cytotoxic’ phenotype. However, based on these data, the study identified 5 clusters in nDNT cells (88): (**i**) Resting DNT cells that feature high expression of killer cell lectin-like receptor D1 (*Klrd1*) (88), a negative regulator of NK cells (92); (**ii**) Helper DNT cells that feature high expression of RAR related orphan receptor A (*Rora)*, a gene that regulates the Th17 lineage in synergy with the RAR related orphan receptor C (*Rorc*) gene (93); (**iii**) Intermediate DNT cells that express Eomesodermin *(Eomes)* (88), a master regulator of cell-mediated immunity capable of controlling the expression of genes encoding effector molecules, such as *Ifng* or *Gzmb* (94); (**iv**) cytotoxic DNT cells with high expression of *Gzmb* (88) and (**v**) innate DNT cells. The significant expression of the *ikzf2* and lymphocyte antigen 6 complex locus C1 (*Ly6c1*) in the resting and “cytotoxic’ DNT cells suggest that IKZF2 may be the critical transcription factor defining the ‘cytotoxic’ DNT phenotype (88). By contrast, the high expression of Ly6c2 in naïve and helper DNT cells suggests that Ly-6C may be a critical gene defining the “resting” DNT phenotype (88). Furthermore, the study showed that ‘cytotoxic’ DNT cells share similar characteristics to CD8^+^T cells (88) and that the naïve and cytotoxic DNT cells shared several highly expressed genes with the activated and resting DNT cells.

**Figure 1:**
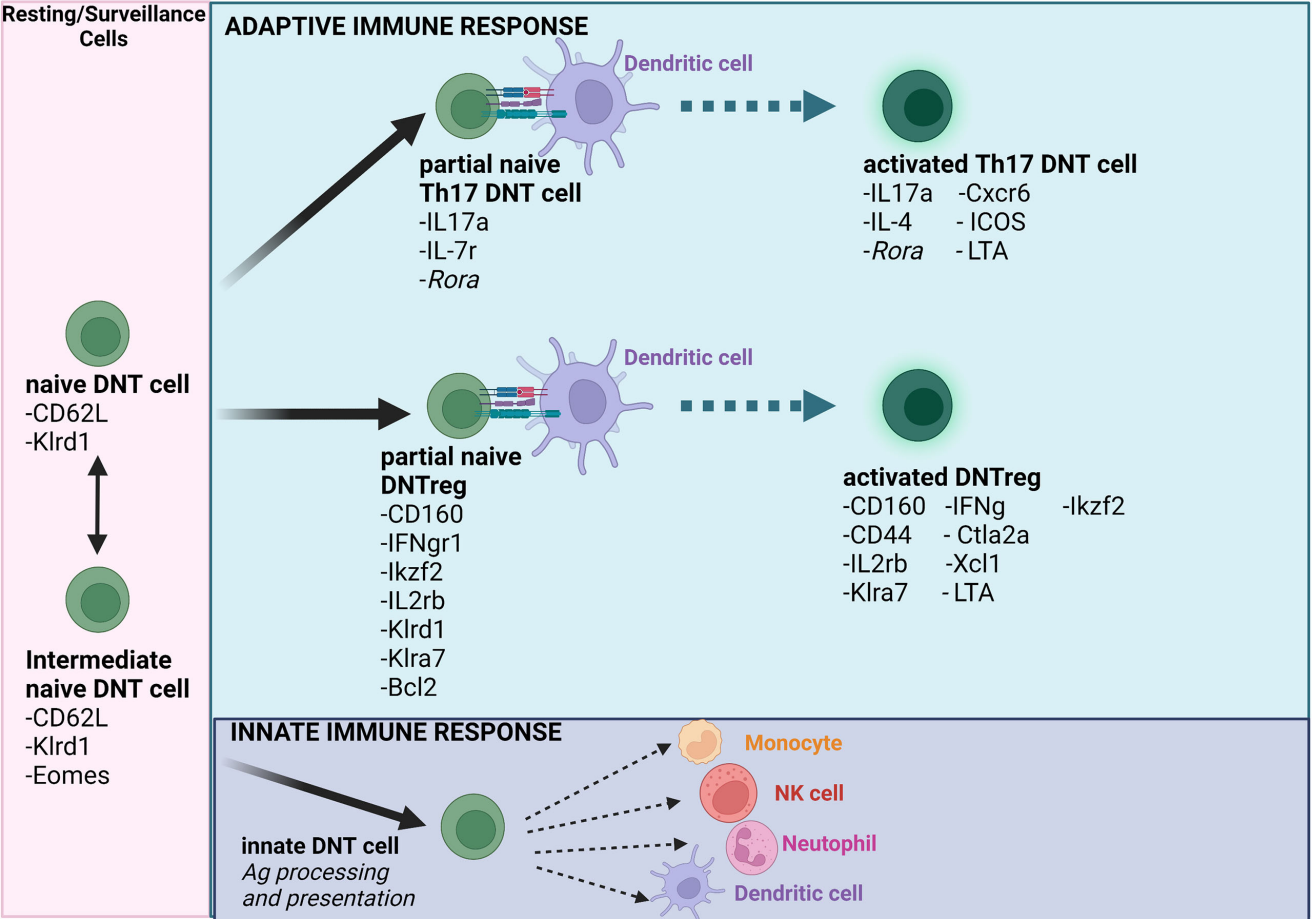
Schematic model of naïve and activated DNT cells. The model summarizes and reflects how transitions impact naïve and activated DNT cells (Note- the literature indicates the possibility of several other DNT cell subsets). Importantly, DNT cells can modulate innate and adaptive immune cells based on immune-modulatory status or the prevailing condition in the tissue microenvironment. Ag = antigen. *Figure created using Biorender.com*.

On activation, the nDNT cells described in the study differentiate to the aDNT groups, with a reduced *Ly6c2* and enhanced *Ikzf2* expression. Though the authors noted that expression levels of *Il17a* were low in aDNT cells, the ‘cytotoxic’ DNT subset that corresponds to DNTregs expressed significantly high levels of cytotoxic genes, including *Gzmb* (88). The aDNT phenotype described in the study has been utilized as DNTreg cellular therapy for acute myeloid leukemic patients indicating that the ‘cytotoxic’ DNT cell phenotype may indeed be DNTregs that can be utilized explicitly as treatments for immune-related diseases (95). Identifying IKZF2 and Ly6C as specific markers of DNTs and possibly DNTregs provides a starting point for further exploration of DNT cells. This study suggests that different subsets of DNT cells are similar to what was proposed in an earlier transcriptomic study on DNT cells from sooty mangabeys (96). The authors reported that DNT cells displayed phenotypes similar to Th1, Tfh, Th2, and Th17 cells (96). It is currently not entirely clear whether DNT cells can differentiate into different subsets or if DNTregs exist as a single phenotype with multiple functions; therefore, further investigations are needed. It is also not entirely clear if IKZF2 specifically identifies the DNTreg subset though it is implied in the study. Perhaps the identification of IKZF2 along with other regulatory surface markers will provide more clarity on DNTreg identification markers. However, based on current literature, it is clear that DNTs can exist as cytolytic or regulatory cells and may also exist in naïve and activated states where the activated states exhibit multiple functions determined by the microenvironment (**Figure 1**). However, this review will primarily focus on the regulatory subtype of DNTs.

Recently, cancer researchers using single-cell RNA sequencing (scRNA-seq) identified 12 clusters of “unconventional” αβTCR^+^ DNT cells in the tumor microenvironment of murine sarcomas supporting the highly heterogeneous nature of DNT cells (97). Furthermore, the study reported that four of these clusters represented 75% of all DNT cells within the tumors, suggesting that the tumor microenvironment can influence the recruitment and differentiation of DNTs. Interestingly, none of the clusters with DNT cells predominantly expressed the checkpoint inhibitors CTLA-4 and PD-1. This finding is supported by a study that showed low CTLA-4 and PD-1 expression in a DNTreg subset (98) but contrasted by another showing high CTLA-4 and PD-1 expression. These cells can also downregulate costimulatory molecules CD80 and CD86 expressed on antigen-expressing mature DCs (mDCs) (99). Though the literature appears inconsistent on DNTregs checkpoint inhibitors’ expression, perhaps expression may depend on the microenvironment. In addition, published data suggest that DNTregs can exert alternative recognition and killing mechanisms through Fas–FasL in an antigen-specific manner (24). The study also suggests that the tissue microenvironment can recruit DNT cells and that these DNT cells adapt their phenotype and function to the prevailing condition. Indeed, several reports support the premise that DNTregs have antigen-specific functions *in vitro* and *in vivo* (24). However, the nature of this function is still under debate since some reports indicate that DNT cells lack major histocompatibility complex (MHC) restriction and have a unique TCR repertoire not derived from positive selection.

Moreover, reports indicate that the antigen-binding characteristics are different from those of MHC-restricted single positive (SP) T cells that bind to antigen epitopes on MHC I or II molecules (100). However, a unique antigen-binding pattern was described for γδTCR DNT cells based on the conformation of the intact antigen and independent of MHC involvement (101). Support for this conclusion comes from a study using mice deficient in both CD4 and CD8 coreceptors and MHC (quad-deficient mice) (102). However, a study proposed that DNTregs can recognize MHC-peptide complexes (103), which can occur through upregulation of the lymphocyte-specific protein tyrosine kinase (Lck), expressed on activation and can drive TCR signal transduction in DNT cells (102). The upregulation of lck orchestrates TCR signaling independent of MHCs (102) and constitutes a straightforward explanation for how the thymus generates MHC-restricted αβTCR. Nonetheless, a study showed that DNT cells could develop independent of lck and mediate potent suppressor functions (104). Since lck mediates TCR interaction with CD8 and CD4 coreceptor molecules (which are absent in DNT cells (105)), other kinases that regulate TCR signaling, such as the proto-oncogene tyrosine-protein kinase (fyn), may therefore be more relevant in driving DNT cell TCR signaling.

## The Female Reproductive System and DNTregs Immune Regulation – Comparison With CD4^+^Tregs

It is well accepted that estrogen drives the expansion of the CD4^+^Treg compartment by inducing their proliferation and promoting suppressive functions (17–19). Interestingly, estrogen also drives the accumulation of DNTs during pregnancy (106). Moreover, like estrogen, seminal fluid also modulates Tregs in the FRS (107–109) and contributes to maternal-fetal tolerance (108, 110).

The roles of CD4^+^Tregs in the FRS, including maternal-fetal tolerance, have been extensively explored and reviewed elsewhere (111–116). We will therefore focus on how DNTregs compare with CD4^+^Tregs in the FRS. CD4^+^Tregs undergo profound changes in the ovary and uterus during the reproductive cycle. In healthy women, an increase is observed during the follicular phase of the menstrual cycle reach maximum numbers during the late follicular phase. This process correlates with increased circulating estrogen levels (11, 117). Moreover, endometrial CD4^+^Tregs expand during the proliferative phase of the cycle (11), which corresponds to the follicular phase in the ovaries. However, as women with reproductive failure experience a dramatic reduction in the population of CD4^+^Tregs (11), it is yet unknown whether a similar phenomenon occurs with DNTregs (117, 118). (119)Since estrogen regulates CD4^+^Tregs and DNTs in the FRS, the function of DNTregs in the FRS may be similar to CD4^+^Tregs. However, establishing precise DNT functions in the FRS and how the populations differ during the female reproductive cycle requires further research. In addition, it is also unknown if seminal fluid modulates DNTreg expansion just as it does CD4^+^Tregs in the FRS and requires further research.

CD4^+^Tregs are also crucial to pregnancy maintenance, as changes to the population of regulatory cells may have severe consequences for fertility (118, 119). Several lines of evidence have described both the systemic and decidual expansion of CD4^+^CD25^+^Foxp3^+^ Treg populations in the first two trimesters of pregnancy (120–122).These findings indicate that CD4^+^Tregs are necessary for reproductive immune tolerance. Since both CD4^+^Tregs and DNTregs have been detected in the non-pregnant endometrium during the reproductive cycle, and reproductive hormones modulate CD4^+^Tregs, we also postulate that similar mechanisms modulate DNTregs during non-pregnant and pregnant conditions. Though the literature on the role of DNTregs in the reproductive system pre- and post-fertilization is scanty compared to that of CD4^+^Tregs, it is clear, based on reports on other tissues, that DNTregs play significant regulatory roles similar to CD4^+^Tregs. Indeed, reports indicate consistently large numbers of DNTregs in the FRS compared to other organs (20, 21, 123). The large numbers of DNTregs found in the uterus, gut, and kidney suggest a preference for highly vascularized regions, particularly mucosal tissues (20, 124). Johansson and Lycke observed that DNTreg numbers in the uterus did not vary in non-pregnant mice, after insemination, and on day-4 pregnancy. They, however, state that this does not exclude a function for these cells during pregnancy (20). That the number of DNTregs did not decrease during early mouse pregnancy may suggest both a relevance or irrelevance for these cells in maintaining maternal-fetal tolerance. A possible explanation for the lack of increase observed during early mouse pregnancy may be related to the finding that during the first trimester, pro-inflammatory cells and cytokines are more relevant as these cells assist the blastocyst to successfully penetrate or ‘implant’ the endometrium (125). However, regulatory cells remain present within the endometrium to keep the pro-inflammatory cells in check and will expand during the second trimester and then decrease during the latter part of the third trimester (125). On the other hand, since CD4^+^Tregs are reported to increase in the first two trimesters of pregnancy (121, 122) and a study reports that DNTregs do not increase or decrease during early mouse pregnancy (20), these findings may also suggest that DNTregs may not play a significant role during early pregnancy and instead may be more relevant as immune response regulators throughout pregnancy duration (and in non-pregnant conditions) (116). However, further studies are required to confirm the role of DNTregs in the different phases of pregnancy. Indeed, a report found that the suppressive capacity of CD4^+^Tregs in peripheral blood and decidua is similar among non-pregnant, pregnant, and patients with recurrent spontaneous abortion (RSA) (121), which could be extrapolated to DNTregs as both cells are recruited and regulated by sex steroids. Moreover, a review by Chapman et al. suggests a possible function of DNTregs during pregnancy which involves maintaining trophoblast and blood vessel integrity and promoting angiogenesis (21).

Taken together, DNTregs can contribute to establishing immune tolerance within the endometrium in preparation for fetal allograft, a concept consistent with their reported suppressive role in preventing rejection of donor-specific allografts (126, 127). While available data on DNTs in different phases of the reproductive cycle are scarce, current knowledge on the presence of DNTs in the FRS and during pregnancy suggests a prominent role for these cells in the FRS that may involve immune regulation and providing support to CD4^+^Tregs which should be explored further.

## DNTregs and CD4^+^Tregs in Tolerance Maintenance - Implications for Reproductive Immune Tolerance

Tolerance to peripheral T cells is necessary to prevent autoimmune damage to self, and regulatory T cells generally act to suppress responses to self and allogeneic antigens (23). Human pregnancy has been described as a condition that requires maintenance of maternal immune tolerance to a semi-allograft fetus comprised of paternally derived antigens. Though the mechanisms involved in sustaining maternal immune tolerance to the semi-allogeneic fetus are still poorly understood, a report suggests that spontaneous abortion is due to the allo-rejection of the fetus by the mother (121). Clearly, a crucial role of regulatory cells in pregnancy involves the mediation of maternal tolerance of the fetus (122). In addition, DNTregs induce donor-specific transplantation tolerance to MHC-mismatched allografts (24). Subsequent reports confirmed that DNTregs induce tolerance to skin and islet transplantation (22, 128), and cardiac xenografts in a donor-specific manner (129) and (122) support a potential role for DNTregs present in the FRS in maintaining reproductive immune tolerance. Studies in several murine models have demonstrated that DNTregs suppress CD4^+^ and CD8^+^ T cell-mediated allogeneic and xenogeneic immune responses and the response to self-antigens (126, 129, 130). DNTregs prevent allograft rejection (131), GvHD (132, 133) and modulate the severity of autoimmune diseases such as autoimmune diabetes (134, 135). However, CD4^+^Foxp3^+^Tregs and human DNTregs appear to require activation by allogeneic antigen-presenting cells (APCs) or anti-CD3/anti-CD28 antibodies to induce their regulatory potential. DNTregs also reversibly suppress the proliferation of responder T cells *via* cell contact-dependent mechanisms (136) which will be discussed subsequently. Furthermore, DNTregs can function as anti-tumor effector cells that mediate nontumor antigen-restricted immunity while maintaining immune regulatory functions (27). These features position DNTregs to effectively function in the different reproductive phases, maintaining remodeling processes and ensuring successful placenta invasion of the endometrium. It is important to note that the precise roles of DNTregs in maternal immune tolerance are yet to be fully established and requires further research. However, we have indicated potential roles of these cells in maternal immune tolerance based on their immune regulatory roles in other tissues and systems.

### Immune-Modulation Mechanisms of CD4^+^Tregs and DNTregs – Implications for Immune Regulation and Tolerance in the FRS

#### Cell-to-Cell Contact and Antigen Specificity of DNTregs-Mediated Suppression

Both CD4^+^Tregs and DNTregs appear to require cell to cell contact to mediate suppression (137–139). CD4^+^Tregs also inhibit other T cells through IL-2 inhibition at the gene transcription level and are not necessarily antigen-specific (138, 140, 141), though *in vivo* antigen-specific suppression can also occur in some subsets (142, 143). However, reports suggest that DNTregs mediate immune suppression in an antigen-specific manner *in vivo* or *in vitro* without competing with other T cells for growth factors or APC surface area. This finding is supported by studies showing that intentionally increasing the APC numbers or the IL-2/IL-4 concentrations did not reverse the DNTreg-induced suppression (24, 139). However, cell-to-cell contact is required for maximal DNTreg mediated suppression, as supernatant from DNTreg clones stimulated *in vitro* with irradiated splenocytes expressing a TCR specific for the *Ld* MHC class I molecule (Ld+) failed to inhibit the naïve anti-Ld response maximally (24). The antigen-specific nature of DNTregs makes these cells excellent tools to be utilized as a targeted novel cellular therapy for reproductive conditions where autoantibodies trigger a self-reactive T cell response to ovarian antigens, such as autoimmune ovarian disease.

#### DNTregs Mediate Immune Suppression of Effector Cells *via* Trogocytosis and TCR Specificity

**Figure 2:**
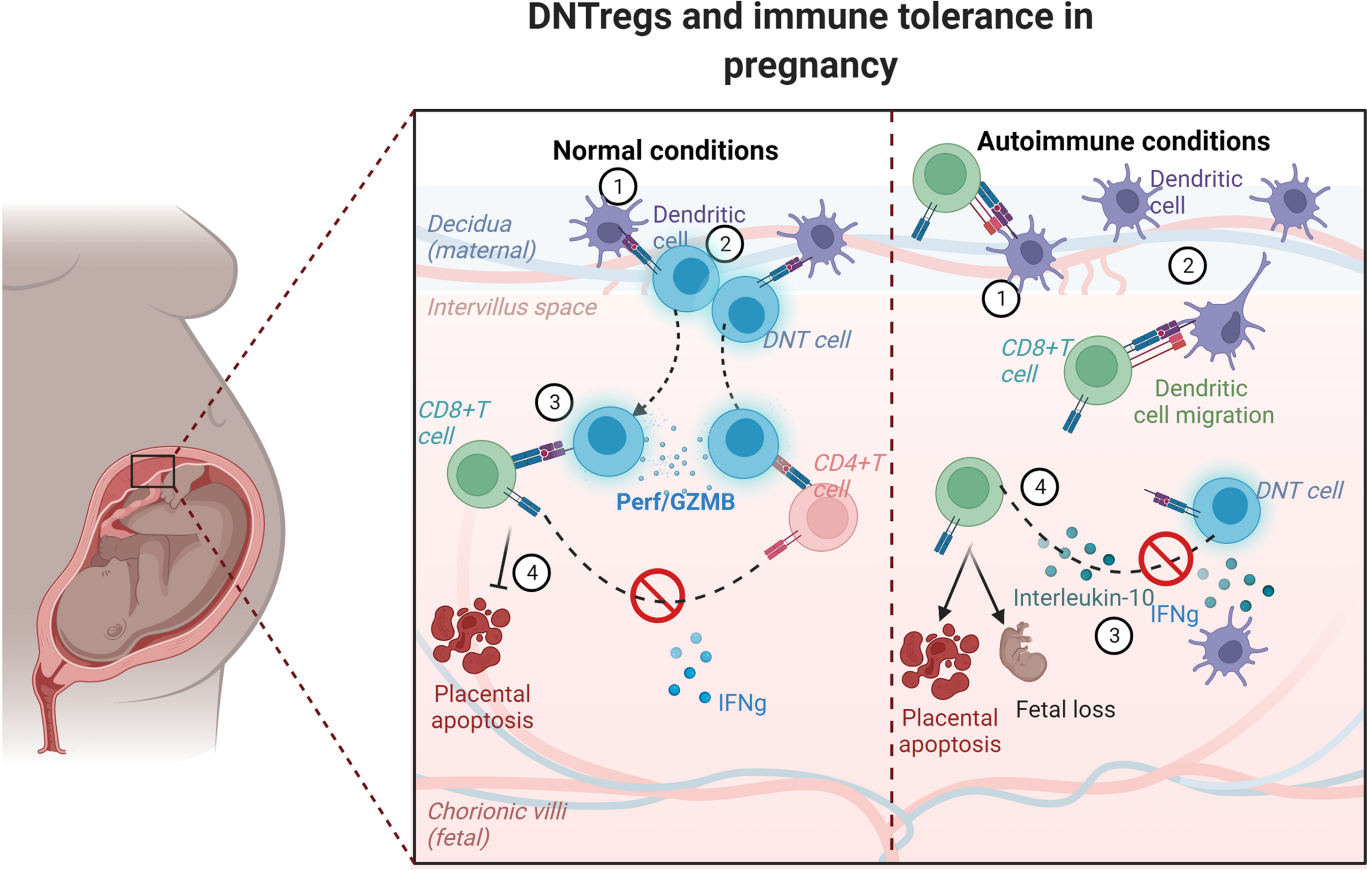
Schematic model showing trogocytosis mechanism by DNTregs in maintaining tolerance in pregnancy. DNTregs utilize trogocytotic mechanisms to suppress recognition of fetal antigens by effector cells and thus prevent placental degeneration and apoptosis in normal physiologic conditions. However, in autoimmune conditions DNTreg populations decrease, and the maternal effectors gain access to the semi-allogenic placenta and induce apoptosis with consequent fetal rejection. The numbers in each panel indicate the sequential progression of interactions between dendritic cells, DNTregs, and effector T cells under normal and autoimmune conditions. Perf = perforin; GZMB= granzyme (B) *Figure created using Biorender.com*.

**Figure 3:**
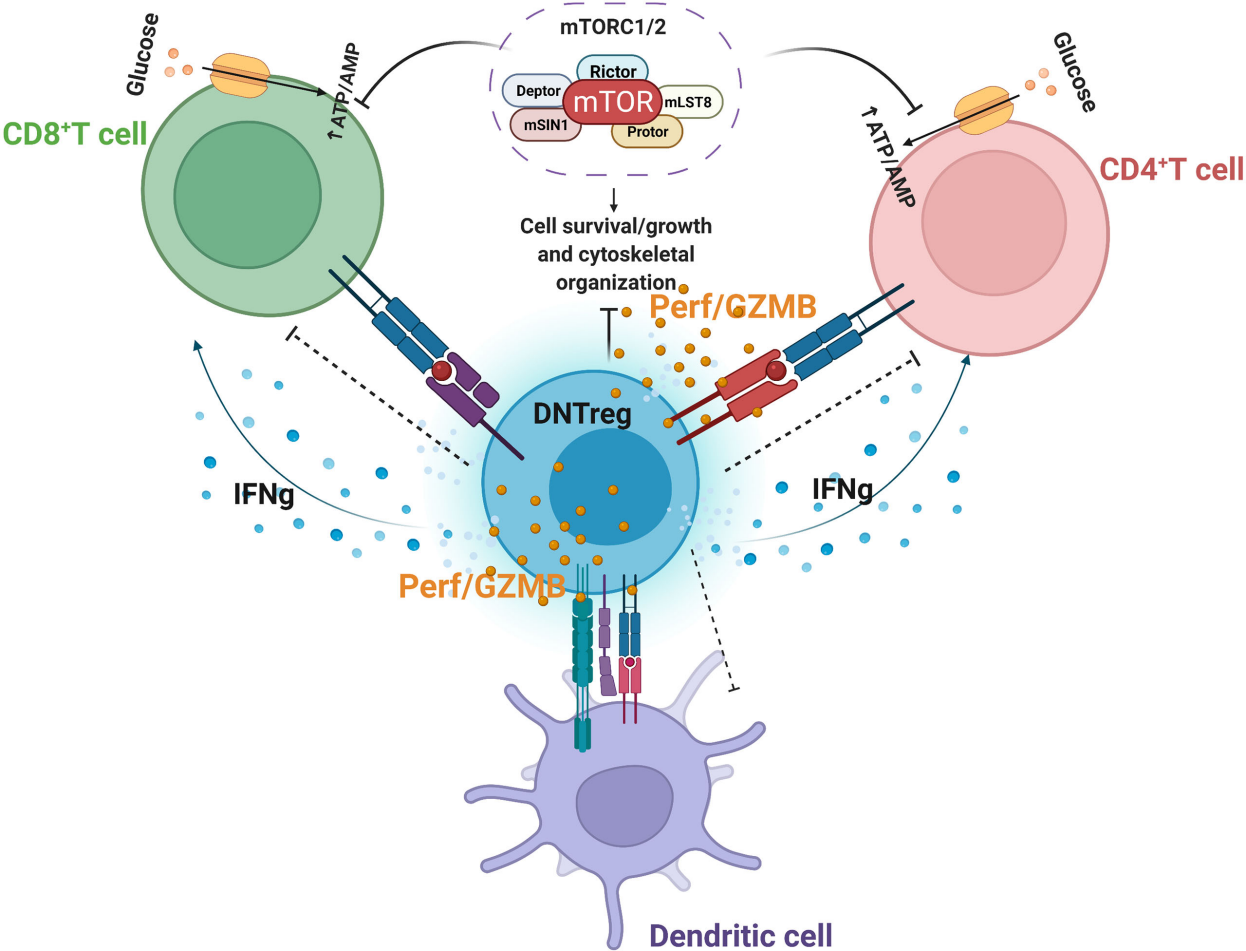
Partial Model of DNTreg suppressor mechanisms. DNTregs can effectively suppress effector CD8^+^, CD4^+^ T cells, and antigen-presenting cells through the antigen-specific trogocytosis mechanism and release of cytolytic proteins, including selective inhibition of mTOR signaling. Perf = perforin; GZMB = granzyme (B) *Figure created using Biorender.com*.

DNTregs present antigens on their surface acquired from APCs, which are recognized by responder T cells bearing TCRs for the cognate antigen (**Figures 2**, **3**). A study showed that DNTregs cells could kill allogeneic and syngeneic CD8^+^T cells that express similar TCR, indicating that a specific TCR interaction may be involved in its suppression mechanism (24). Thus, it would seem that a shared TCR-specificity between DNTregs and target T cells may be one of the factors required for cytotoxicity to occur. Furthermore, reports indicate that DNTregs may not participate in bystander killing (24). Just as DNTregs require stimulation through their TCR to gain a suppressive phenotype, CD4^+^Tregs also require TCR stimulation for their function. CD4^+^ Treg TCR stimulation can be mediated *in vitro* through specific peptides but not through third-party antigens (140, 144). Murine and human DNTregs have a unique feature of retaining surface expression of the acquired molecules for several days (24), providing a wide window to enhance the magnitude of T cell suppression.

Interestingly, studies found that DNTregs-mediated suppression can be attenuated by blocking the TCR expression of responder T cells or blocking the DNTreg-acquired antigen, a mechanism associated with trogocytosis (24). Trogocytosis is a unique process utilized by some immune cells to acquire proteins *via* active transfer from neighboring cells (145–148). The term ‘trogo’ is obtained from the ancient Greek meaning ‘to gnaw’ (146). Although CD4^+^Tregs utilize trogocytosis to acquire peptides from APCs as a means of suppression (149), those cells do not present antigens to effector T cells like DNTregs. Moreover, the lack of CD4 or CD8 coreceptors (CD4 or CD8) and the often low to moderate expression of CD28 (22, 75) suggest that DNTregs utilize other costimulatory molecules for their activation after trogocytosis.

Clinical trials of TCR-engineered T cells produce better responses than T cells genetically modified to express chimeric antigen receptors (CAR-T) due to the high TCR specificity achieved (150). Furthermore, that DNTregs uniquely target allogeneic or syngeneic T cells with the same TCRs indicates a high degree of sensitivity and specificity that can be utilized to manage autoimmune reproductive disorders (**Figure 3**).

#### DNTregs: Mechanisms of Suppression and Cytokine Regulation

DNTregs and CD4^+^Tregs express unique cell surface markers promoting suppressive functions. On activation, DNTregs (like CD4^+^Tregs) express the T cell early activation markers CD25 and CD69 (24), and on TCR ligation, DNTregs do not shed CD62L (24). Though it was earlier reported that DNTregs do not express the activation markers CD44 or CD28 after activation (24), a transcriptomic study on DNTregs showed that DNTregs express the CD44 activation marker (88) similar to CD4^+^Tregs (151). Clearly, DNTregs share some similarities with Tregs as both cells expand in the presence of exogenous IL-2 and IL-4 (24), and on activation, they both express TCR (which may be αβ or γδ for DNTs (21, 24, 83)), CD45, CD25, LFA-1, CTLA-4, CD69, and CD62L (24, 99). Whether DNTregs exclusively express TCRαβ or whether DNTregs exist as subsets within both TCRαβ and γδ expressing DNT cells is not quite unclear. Several studies show that DNTs expressing TCRαβ do exhibit immunoregulatory potential (20, 24, 136), while another study show same cells participate in anti-tumor activities (76). However, that DNTregs exhibit anti-tumor activity while retaining immunoregulation function appear to be a unique function of DNTregs (27) as discussed earlier in this review. In addition, some articles suggest that DNTs positive for TCRγδ exhibit immunoregulatory activities (21, 83). Further investigations are therefore recommended to confirm the TCR subtypes expressed by DNTregs. However, unlike CD4^+^Tregs, human and murine DNTregs lack Foxp3 expression (22, 99, 136). DNTregs also express unique cytokines that are slightly different from CD4^+^Tregs, Th1, Th2, Th17, or Th3 cells. While CD4^+^Tregs release anti-inflammatory cytokines such as IL-4, IL-10, TGF-β, or IL-35 (152–156), reports indicate that DNTregs constitutively secrete IFNg, TNF-α, and insignificant amounts of TGF-β (24) (**Figures 1**, **3**). Secretion of IL-10 by DNTregs is still under debate as one study reports that DNTregs do not secrete IL-10 (24), and other studies indicate secretion of IL-10 by DNTregs (96, 124, 157). The experimental conditions utilized by the different studies may have modulated the utilization of IL-10 by DNTregs, but this remains to be clarified. That human and mice DNTregs express high levels of IFNg supports a variety of modulatory functions beyond its reported killing ability. Perhaps IFNg production by DNTregs provides regulatory functions and enhances their cytolytic activities in active inflammatory environments, such as autoimmune reproductive failure or pregnancy disorders. Support for this possibility comes from studies that show a requirement by CD4^+^Tregs for IFNg to promote suppression of allogeneic immune responses (158, 159). In addition, these findings are supported by a report that CD4^+^Tregs in the maternal-fetal interface produce IFNg, which appear to equip these cells to influence immune responses through several molecular pathways and cellular targets (160). That DNTregs produce IFNg as a primary cytokine supports a multifunctional capacity necessary for modulating tolerogenic and autoimmune conditions.

Like CD4^+^Tregs, DNTregs require exogenous IL-2 for proliferation both *in vitro* and *in vivo* (24, 161, 162). DNTregs can also be activated and expanded *in vitro* by allogeneic splenocytes in the presence of exogenous IL-2, and IL-4, where IL-4 protects DNTregs from TCR-crosslinking induced apoptosis (163). It is, however, unclear whether cytokines known to activate Tregs, such as IL-4 or IL-15 (164), could also activate DNTregs. These findings support that DNTreg suppression combines direct and indirect suppression mechanisms observed with CD4^+^Tregs. Taken together, it appears that DNTregs possess a unique array of regulatory proteins and cytokines that differ from Th1, Th2, or Treg cells (165, 166).

#### DNTregs Mediate the Killing of Target Cells *via* Cytotoxic and Cytolytic Mechanisms

Two major pathways are involved in T-cell-mediated cytotoxicity: perforin-dependent and Fas-dependent pathways (167). Though both activated regulatory and non-regulatory T cells express similar levels of TCR and Fas ligand (FasL) (163), target T cells can be protected from DNTreg-mediated killing through low or absent functional Fas receptors (130). However, studies showing that DNTregs can directly kill target T cells through Fas/FasL interactions (24) also hint that the Fas/FasL mechanism is not the sole killing mechanism by DNTregs. Indeed, studies show that blocking FasL with Fas-Fc does not entirely prevent the killing ability of DNT cells and that DNTreg’s ability to suppress proliferation is more potent than their cytotoxic ability (61, 128, 168). Moreover, in the absence of cell-to-cell contact, DNTregs appear to mediate minimal suppression (61, 168). Specifically, reports show that DNTregs secrete perforin (75, 169) and granzyme proteins (169), a feature shared by naturally occurring CD4^+^Tregs (170, 171), supporting the hypothesis that DNTreg-mediated killing goes beyond the Fas/FasL pathway (145).

Recent evidence suggests that DNTregs are resistant to apoptosis induction *in vitro* and *in vivo*, as DNTregs did not undergo significant apoptosis on TCR cross-linking compared to conventional T cells (172). Furthermore, a report stated that DNTregs persist for more prolonged periods than CD8^+^ T cells after infusion into alloantigen expressing mice (132), suggesting the resilience of DNTregs to activation-induced cell death. This feature may allow DNTregs to function for prolonged periods and increase immune regulation *in vivo*. Nonetheless, a study describes that incubation of DNTregs *in vitro* with IL-10, abolishes their ability to resist apoptosis, and diminishes their suppressive function (152). These findings suggest that the Th1/Th2 cytokine balance plays a central role in modulating DNTregs’ function *in vivo*. However, that reports show activated DNTregs to secrete IL-10 (60, 157) opens more questions regarding the role of IL-10 on DNTregs; for instance, does IL-10 reduce the survival potential of DNTregs or is there an IL-10 threshold requirement that balances function and apoptosis? Further studies are needed to evaluate the role of IL-10 on the function of DNTregs as modulating IL-10 secretion by DNTregs may prove helpful in enhancing their suppressive activity in patients with autoimmune diseases, including in autoimmune reproductive disorders when utilized as a cellular therapy.

#### DNTregs Mediate Metabolic Suppression of Effector T Cells

A recent finding indicated that in addition to Fas/FasL mechanisms and cytolytic protein release, DNTregs mediate suppression in effector T cells by inhibiting the selective mammalian target of rapamycin (mTOR) (173). The mTOR enzyme induces metabolic reprogramming of alloantigen activated T-cells after allogeneic hematopoietic stem cell transplantation, which utilizes the glycolytic pathway to sustain alloreactive T cells mediated graft versus host disease (GvHD) (174, 175). In the presence of DNTregs, downstream mTOR signaling pathways modulated by effector T-cells are abolished (173). Inhibitory molecules, such as PD-1 and CTLA-4, can target and terminate mTOR phosphorylation and metabolic reprogramming of T cells by engaging distinct phosphatases (174, 176). DNTregs can therefore utilize the expression of checkpoint inhibitory molecules such as CTLA-4 or PD-1 to engage effector T cells and target their mTOR signaling pathway. DNTregs also inhibit mTOR-mediated increases in transcription factor HIF-1α without affecting NF-ƙB activation and p38 pathways (173). Furthermore, DNTregs selectively downregulate glucose transporters (GLUT1 and 3) in effector T cells and reduce the glycolysis capacity without interfering with their fatty acid uptake (173) (**Figure 3**). This selective trait is similar to PD-1 mechanisms where T-cell glycolysis is inhibited, and fatty acid oxidation proceeds uninterrupted (177).

While CD4^+^Tregs also interfere with effector cell metabolism, it occurs through pathways other than those targeted by DNTregs which include (**i**) expression of CD39, and CD73 nucleases for hydrolysis of extracellular ADP or ATP into AMP and adenosine (178), (**ii**) competition for IL-2 required for proliferation of CD4^+^Tregs (179), (**iii**) through the transfer of cAMP to effector T cells and (**iv**) through IL-27 signaling that upregulates CD39 in CD4^+^Tregs (180). However, it is currently unknown if DNTregs express CD39 or CD73 and further research is required to examine these possibilities.

#### DNTregs Alter the Phenotype and Migratory Capacity of Effector T Cells

*In vitro* studies show that DNTregs inhibit the induction of the transcription factor T-bet in activated effector T cells without interfering with the expression of Eomes (173). Regulation of transcription factors like T-bet and Eomes modulates differentiation of memory and effector T cells, and mTOR signaling plays a significant role in regulating T-bet and Eomes (181). A study detected elevated levels of Eomes in activated effector T cells co-cultured with DNTregs, and a corresponding reduction of T-bet expression (173). The authors also showed that Foxp3 expression (which is also a transcription factor) was not enhanced in the presence of DNTregs (173). Since transcription factors orchestrate the expression of distinct T cell markers, DNTregs regulate the transcription factor activity in effector T cells by suppressing upregulation of the costimulatory cell surface molecule CD28. Interestingly, expression of the costimulatory receptor CD27 was not reduced but further enhanced in the presence of DNTregs (173). DNTregs also alter the phenotypic chemokine expression of effector T cells. In the presence of DNTregs, CD4^+^T cells become CD45RO^+^ and CCR7^+^ with enhanced expression of CD27 and CXCR5 (173). Since the phenotype of naive T cells is CCR7^+^ and CD45RO^-^, while that of effector T cells are CD45RO^+^ and CCR7^-^ (182), this indicates that DNTregs are capable of switching effector T cells to long-living central-memory T-cells characterized by CCR7, CD27, and CXCR5 expression (183–185). It also indicates that DNTregs facilitate the trafficking of effector T cells away from inflammation sites and increase homing to lymphoid organs. It may therefore be that DNTreg-induced mTOR inhibition mediates these phenotypic alterations through other as yet unknown mechanisms. Suppression of T-bet activity invariably suppresses the expression of pro-inflammatory chemokine receptors such as CXCR3 and CCR5, which are necessary for the migration and infiltration of effector T-cells to their target tissue (186, 187). Since coordinated migration of cells by chemokine receptors is required to execute T cell effector function appropriately, DNTregs present another mechanism to interfere with T cell function in autoimmune reproductive disorders.

While the pattern of DNTreg selective modulation of transcription factors is still unclear, DNTregs decrease naïve T cell phenotype and induce a long-living central-memory T-cell phenotype. The induction of long-living central memory T cells reduces activation and re-activation of effector T cells and can be exploited for tolerance maintenance in reproductive disorders. In addition, DNTregs promote and sustain suppressive T cell phenotypes, which is a relevant trait for fertility success.

#### DNTregs Modulate Effector T Cell Functions

Though activated effector CD4^+^T cells secrete significant amounts of IFNg, in the presence of DNTregs, IL-2 expression was not concurrently increased. Instead, decreased production of IFNg, IL-17a, and granulocyte-macrophage-colony-stimulating factor (GMCSF) was observed in the presence of DNTregs (173). The ability of DNTregs to reduce the secretion of pro-inflammatory cytokines by effector T cells could have important implications for pregnancy maintenance. Intriguingly, DNTregs enhanced CD4^+^ T cells’ IL-2 production; however, the authors explain that though IL-2 is required for T cell proliferation and survival, it can also selectively restore CD4^+^Foxp3 Tregs immunosuppressive function without activation of T cells (188). In comparison, CD4^+^ Foxp3^+^ Tregs induce senescence in effector T cells (189), but DNTregs do not.

Furthermore, CD4^+^Foxp3^+^Tregs do not modulate chemokines and cytokines that DNTregs are shown to induce. In addition, DNTregs may potentially support Foxp3^+^ Tregs after adoptive transfer by enhancing T cell IL-2 production. Juvet and Zhang. 2012 also proposed the possibility of a functional interaction between DNTregs and CD4^+^Tregs. The authors support their arguments through reports from a study that showed a significantly increased CD4^+^CD25^+^Foxp3^+^Treg population and extended cardiac allograft survival on adoptive transfer of DNTregs (190). In addition, a study on sooty mangabeys which report that DNT cells can contribute to preserving CD4^+^T cells during chronic infections (96) supports this possibility. If this indeed is the case, increasing the numbers of DNTregs in autoimmune reproductive pathologies through adoptive transfer will provide direct control of immune responses while simultaneously potentiating the response and function of CD4^+^Tregs. DNTregs prove a promising regulatory subset of T cells that abolishes and modulates target cell function. These findings reveal interesting new targets through which DNTregs can selectively modulate effector T cells’ signaling and metabolic programming and pave the way for the utilization of DNTregs as a targeted cellular therapy for reproductive failure and pregnancy disorders.

**Figure 4:**
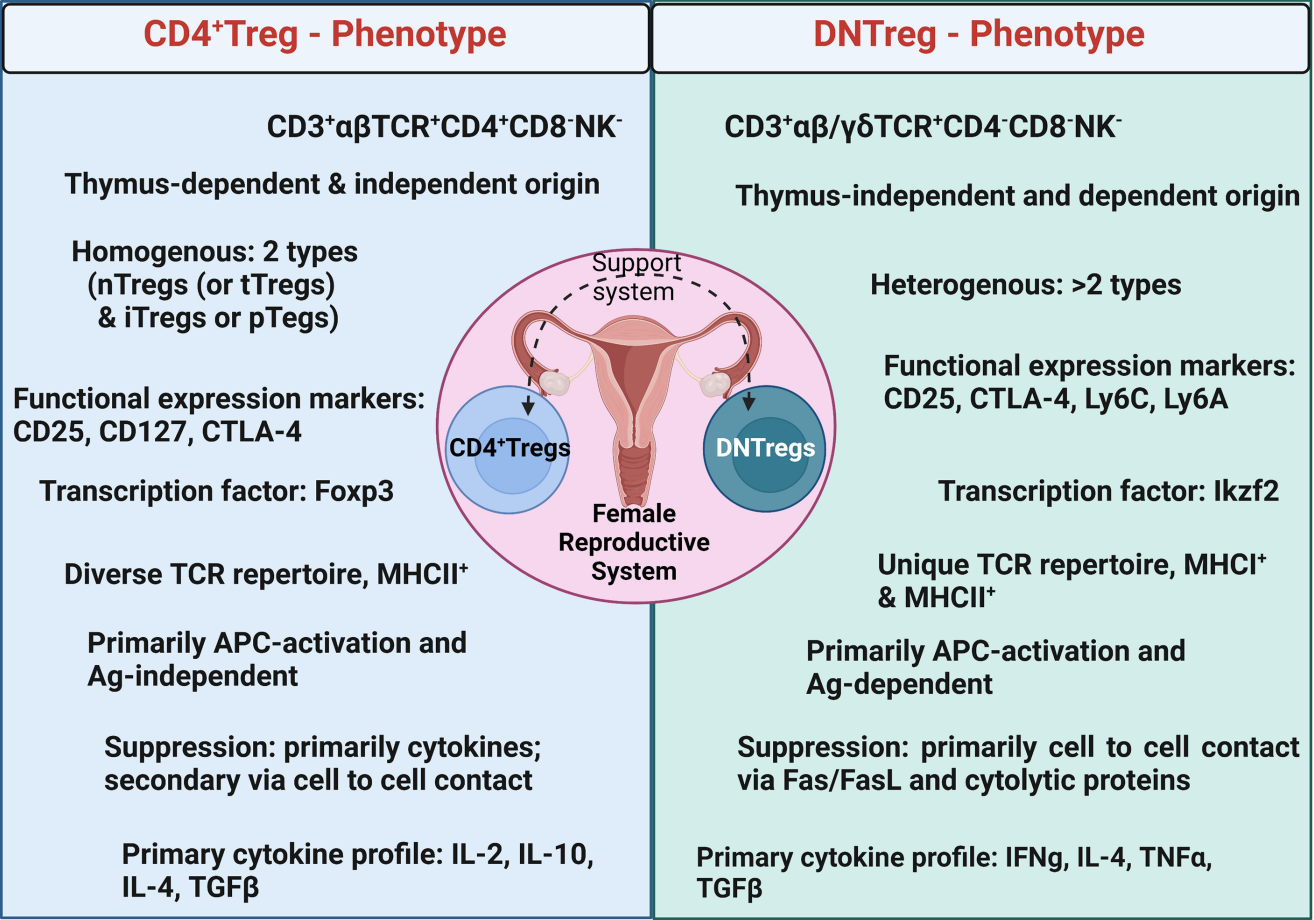
Summary of major phenotypic characteristics of DNTregs compared to CD4^+^Tregs. Ag = Antigen; nTregs= naturally occurring Tregs; tTregs= Tyhmic Tregs; iTreg = inducible Tregs; pTregs = peripheral Tregs. *Figure created using Biorender.com*.

In summary, DNTregs are a regulatory subset of T cells with distinct functions and cytokine expression unique to the prevailing conditions of the tissue environment (**Figure 4**). Furthermore, such plasticity of DNTregs allows unique adaptive capacity to the rapidly changing and variable physiologic conditions within the FRS. Therefore DNTregs are poised to become novel therapeutic tools for FRS pathologies.

## Conclusion

The influx of immune cells into the FRS at different cycle stages, particularly during the ovulatory and endometrial process, indicates the importance of immune cells with regulatory functions in maintaining tolerance from pre-conception to implantation and pregnancy (119, 191, 192). While the information on DNTregs within the FRS is still growing, this review highlights the capacity and functions of DNTregs, a unique and remarkable regulatory cell subset complimentary to CD4^+^Tregs, and their potential in supporting FRS functions. DNTregs possess unique features for enhanced specificity, plasticity, effective killing, and suppression while evading hostile microenvironments. These features and the findings that DNTregs preferentially home to mucosal sites and are additionally dominant in the FRS, position DNTregs as novel regulators with extraordinary potential to become the next targeted cellular therapy, particularly for autoimmune female reproductive disorders. That DNTregs can be successfully expanded from healthy individuals and utilized as an off-the-shelf therapy enhances their relevance and provides an emerging shift in considering regulatory cell subtypes for autoimmune conditions. *via*

## Author Contributions

EB conceptualized and wrote the article; JV participated in writing and reviewing the article; HY participated in conceptualizing, reviewing, and writing the article. All authors contributed to the article and approved the submitted version.

## Conflict of Interest

The authors declare that the research was conducted in the absence of any commercial or financial relationships that could be construed as a potential conflict of interest.

## Publisher’s Note

All claims expressed in this article are solely those of the authors and do not necessarily represent those of their affiliated organizations, or those of the publisher, the editors and the reviewers. Any product that may be evaluated in this article, or claim that may be made by its manufacturer, is not guaranteed or endorsed by the publisher.
